# Ultrasound-Guided Intertransverse Process and Deep Rectus Sheath Blocks for Emergency Laparoscopic Cholecystectomy in a High-Risk Elderly Patient: A Case Report

**DOI:** 10.7759/cureus.114236

**Published:** 2026-08-09

**Authors:** Tommaso Sorrentino, Francesco Marrone, Pierfrancesco Fusco, Giovanni Cosco, Pasquale Castaldo, Marco Cannistrà, Sam Mahli, Monica Muratgia, Giusy Milone, Angelo Gigliarano, Andrea Scardaci, Fabrizio Fattorini

**Affiliations:** 1 Anesthesia, San Giovanni di Dio Hospital Crotone, Crotone, ITA; 2 Anesthesia and Intensive Care, Santo Spirito Hospital, Rome, ITA; 3 Anesthesia and Intensive Care Unit, San Filippo e Nicola Hospital, Avezzano, ITA; 4 Surgery, San Giovanni di Dio Hospital Crotone, Crotone, ITA; 5 Medical and Surgical Science, Anesthesia and Intensive Care, Magna Graecia University, Catanzaro, ITA; 6 Anesthesiology, San Sebastiano Hospital, Frascati, ITA

**Keywords:** dexmedetomidine, fascial plane blocks, laparoscopic cholecystectomy, peritonitis, regional anesthesia

## Abstract

Gangrenous cholecystitis with peritonitis in elderly, high-risk patients carries substantial perioperative risk, particularly when compounded by multiple comorbidities, coagulopathy, and sepsis. General anesthesia in such patients is associated with increased hemodynamic instability and postoperative morbidity. Regional anesthesia combined with procedural sedation has been proposed as a safer alternative in selected high-risk surgical candidates. We report the case of an 81-year-old woman who presented with cholecystitis complicated by diffuse peritonitis. Her medical history included cerebral ischemic stroke, diabetes mellitus, stage 3 chronic kidney disease, and arterial hypertension. On admission, she was septic, with elevated C-reactive protein (44 mg/L) and procalcitonin (10.9 ng/mL), hypoalbuminemia (2.5 g/dL), and a deranged coagulation profile (INR 1.9). Given the high anesthetic risk associated with general anesthesia, a regional strategy was adopted, combining an ultrasound-guided intertransverse process (ITP) block (T7-T8/T8-T9, ropivacaine 0.2%, 20 mL) with bilateral deep rectus sheath (DRS) blocks (ropivacaine 0.2%, 20 mL each side). Surgery began 30 minutes after block placement under sedation with dexmedetomidine (1 mcg/kg bolus followed by 1.2 mcg/kg/h infusion) and ketamine (0.25 mg/kg/h, total dose 40 mg). Laparoscopic cholecystectomy was completed in 90 minutes, with hemodynamics remaining stable throughout (mean arterial pressure approximately 65 mmHg). The patient emerged from the operating room sedated but breathing spontaneously, without the need for airway instrumentation, and was admitted to the intensive care unit for monitoring. She was discharged from the intensive care unit after 20 hours in stable condition and discharged home on postoperative day 8. Combined ultrasound-guided ITP and DRS blocks, supplemented with dexmedetomidine-ketamine sedation, provided effective anesthesia for emergency laparoscopic cholecystectomy while avoiding general anesthesia in an elderly, septic, high-risk patient with coagulopathy. This approach may represent a viable strategy to reduce perioperative risk in similarly frail surgical candidates.

## Introduction

Laparoscopic cholecystectomy performed as an emergency procedure in frail, elderly patients poses significant challenges, particularly when multiple comorbidities make general anesthesia a high-risk choice. Neuraxial anesthesia is a viable alternative to general anesthesia for laparoscopic cholecystectomy, with a systematic review and meta-analysis reporting comparable outcomes between the two approaches [1]. Segmental thoracic spinal anesthesia in particular has been described for this procedure in selected high-risk patients, using low doses of local anesthetic injected at the thoracic level to reduce hemodynamic impact and shorten the duration of the motor block [2,3]. However, such an approach may not be feasible in patients with spinal abnormalities and, particularly, in those with a deranged coagulation profile, which represents a contraindication to central neuraxial techniques. Bilateral thoracic paravertebral blocks have been shown to provide analgesia non-inferior to thoracic epidural analgesia for abdominal surgery requiring midline laparotomy, when used as part of a multimodal regimen [4].

Nevertheless, paravertebral blocks are considered deep blocks and are therefore contraindicated in the case of coagulopathy. As an alternative, fascial plane blocks have gained increasing support as components of multimodal analgesia for major abdominal surgery. Additionally, the combined use of erector spinae plane (ESP) and intertransverse process (ITP) blocks, both regarded as paravertebral-by-proxy techniques, has been described as a primary anesthetic strategy in high-risk patients undergoing open gastrectomy [5] and upper abdominal laparoscopic surgery [6], thereby avoiding airway instrumentation and general anesthesia.

Here, we report the use of a combined ITP and deep rectus sheath (DRS) block approach as the primary anesthetic technique for emergency laparoscopic cholecystectomy in a high-risk patient, in whom general anesthesia posed substantial risk and spinal anesthesia or paravertebral blocks were not feasible due to coagulopathy.

Our strategy was built on two components: the ITP block, a “paravertebral-by-proxy” block that, in terms of injection plane depth, lies halfway between the ESP block and the true thoracic paravertebral block, providing potentially effective visceral analgesic coverage without the need to reach the actual paravertebral space. The second component was the DRS block, a more recently described technique that provides preperitoneal analgesia, acting on the parietal peritoneum. Its analgesic efficacy in laparoscopic cholecystectomy has been documented both in previous reports and in a recent randomized trial [7,8].

The manuscript adheres to the CARE (Consensus-Based Clinical Case Reporting) guidelines for reporting case reports as per EQUATOR (Enhancing the Quality and Transparency of Health Research) guidelines. Written informed consent was obtained from the patient for the publication of this case report and accompanying images.

## Case presentation

We present the case of an 81-year-old woman (weight 75 kg, height 160 cm, body mass index 29.3 kg/m²) who presented to the emergency department with gangrenous cholecystitis complicated by peritonitis and evolving septic status. Her past medical history was significant for cerebral ischemic stroke, non-insulin-dependent diabetes mellitus, stage 3 chronic kidney disease, and arterial hypertension.

On clinical examination, her general condition was markedly compromised. Chest radiography revealed a right-sided pleural effusion associated with respiratory insufficiency. Laboratory investigations demonstrated a severe inflammatory response, with elevated white blood cell count, C-reactive protein (CRP), and procalcitonin (PCT), consistent with septic status. Additional findings included hypoalbuminemia and a deranged coagulation profile, with an elevated international normalized ratio (INR) (Table 1).

**Table 1:** Laboratory investigations on admission WBC: white blood cell count, CRP: C-reactive protein, PCT: procalcitonin, INR: international normalized ratio

Parameter	Value	Unit	Reference
WBC	12,300	/µL	4,000-11,000/µL
CRP	44	mg/L	<5 mg/L
PCT	10.9	ng/mL	<0.5 ng/mL
Albumin	2.5	g/dL	3.5-5.0 g/dL
INR	1.9	-	0.8-1.2

The patient was classified as American Society of Anesthesiologists (ASA) physical status IV. Preoperative risk stratification using the American College of Surgeons National Surgical Quality Improvement Program (ACS NSQIP) Surgical Risk Calculator confirmed a markedly elevated perioperative risk profile compared to the average patient undergoing the same procedure, with predicted risk of a serious complication of 24%, 30-day mortality of 10%, and a predicted length of hospital stay of six days [9].

A multidisciplinary discussion involving the anesthesiology and surgical teams was undertaken to define the safest anesthetic strategy. General anesthesia was considered high-risk given the patient's age, septic status, respiratory compromise, and overall frailty. Paravertebral blocks and neuraxial techniques, although potential alternatives for laparoscopic cholecystectomy, were considered contraindicated because of the deranged coagulation profile, in addition to the patient's advanced age and possible spinal degenerative changes. After thorough discussion of the risks associated with general anesthesia and the non-feasibility of neuraxial techniques, written informed consent was obtained from the patient for a combined locoregional anesthetic approach, with general anesthesia maintained as a contingency plan in the event of block failure or surgical necessity.

Following standard monitoring (ECG, invasive blood pressure, and pulse oximetry), a combined ITP and DRS block was performed under strict aseptic technique and real-time ultrasound guidance (SonoSite PX; FUJIFILM Sonosite, Inc., Bothell, WA, USA).

**Figure 1:**
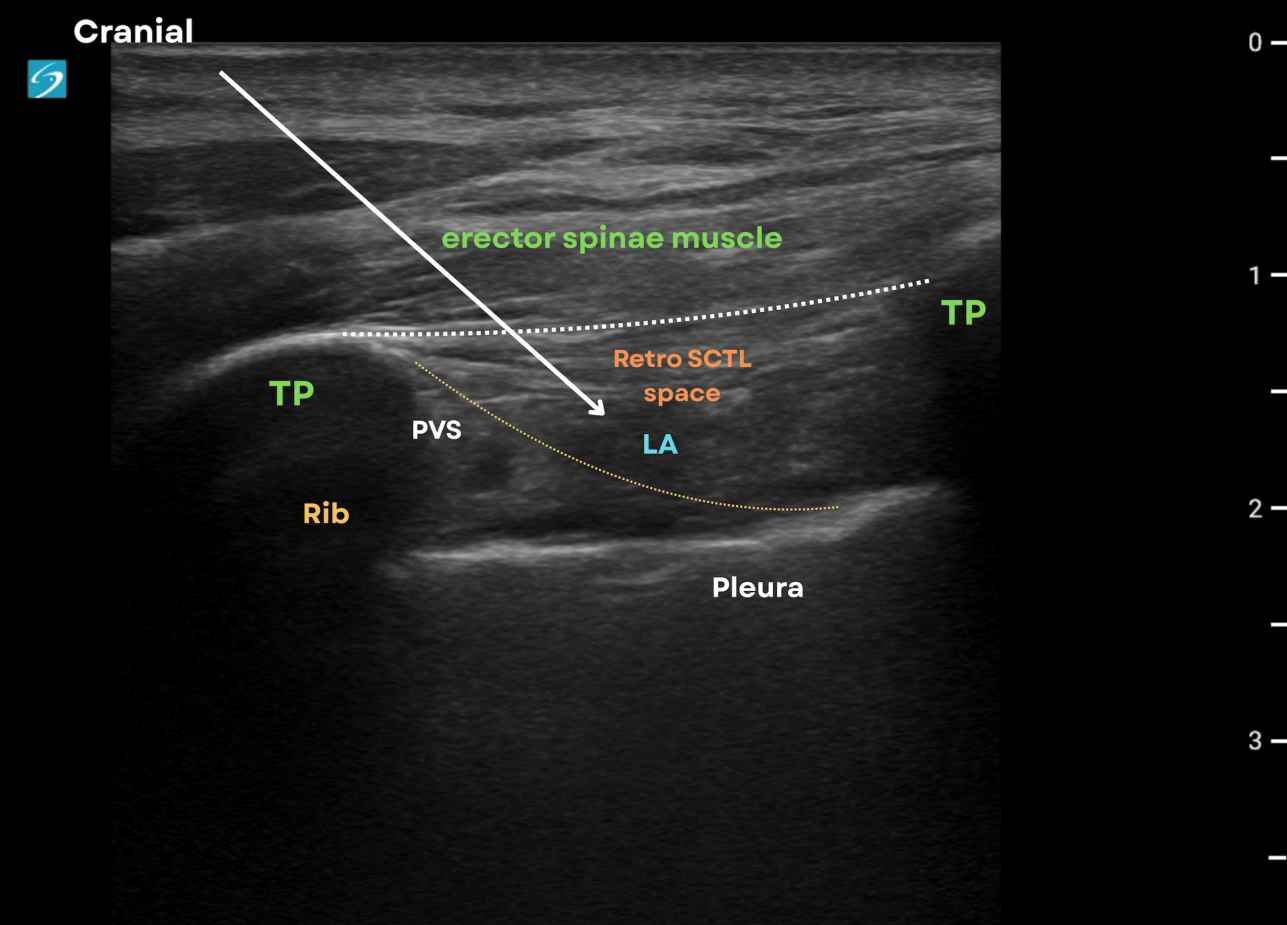
ITP block The dotted yellow line represents the SCTL. The white arrow indicates the needle trajectory toward the retro-SCTL space, immediately to the SCTL, injecting the LA outside the PVS. The white dotted line represents the intertransverse ligament, the fascial plane under the erector spinae muscle, between two adjacent TPs, which is targeted in the ESP block. Ultrasound orientation markers are shown within the image (cranial to the left, caudal to the right; superficial and posterior at the top, deep and anterior at the bottom). SCTL: superior costotransverse ligament, LA: local anesthetic, PVS: paravertebral space, TPs: transverse processes, ITP: intertransverse process, ESP: erector spinae plane

The unilateral ITP block was performed with the patient in the lateral decubitus position, using a high-frequency linear probe positioned longitudinally over the T7-T8 and T8-T9 transverse processes on the right side. The needle was advanced in-plane, in a craniocaudal direction, targeting the tissue plane between two adjacent transverse processes, above the superior costotransverse ligament. A total of 20 mL of ropivacaine 0.2% was administered, with visualization of local anesthetic spread posteriorly to the superior costotransverse ligament (Figure 1).

**Figure 2:**
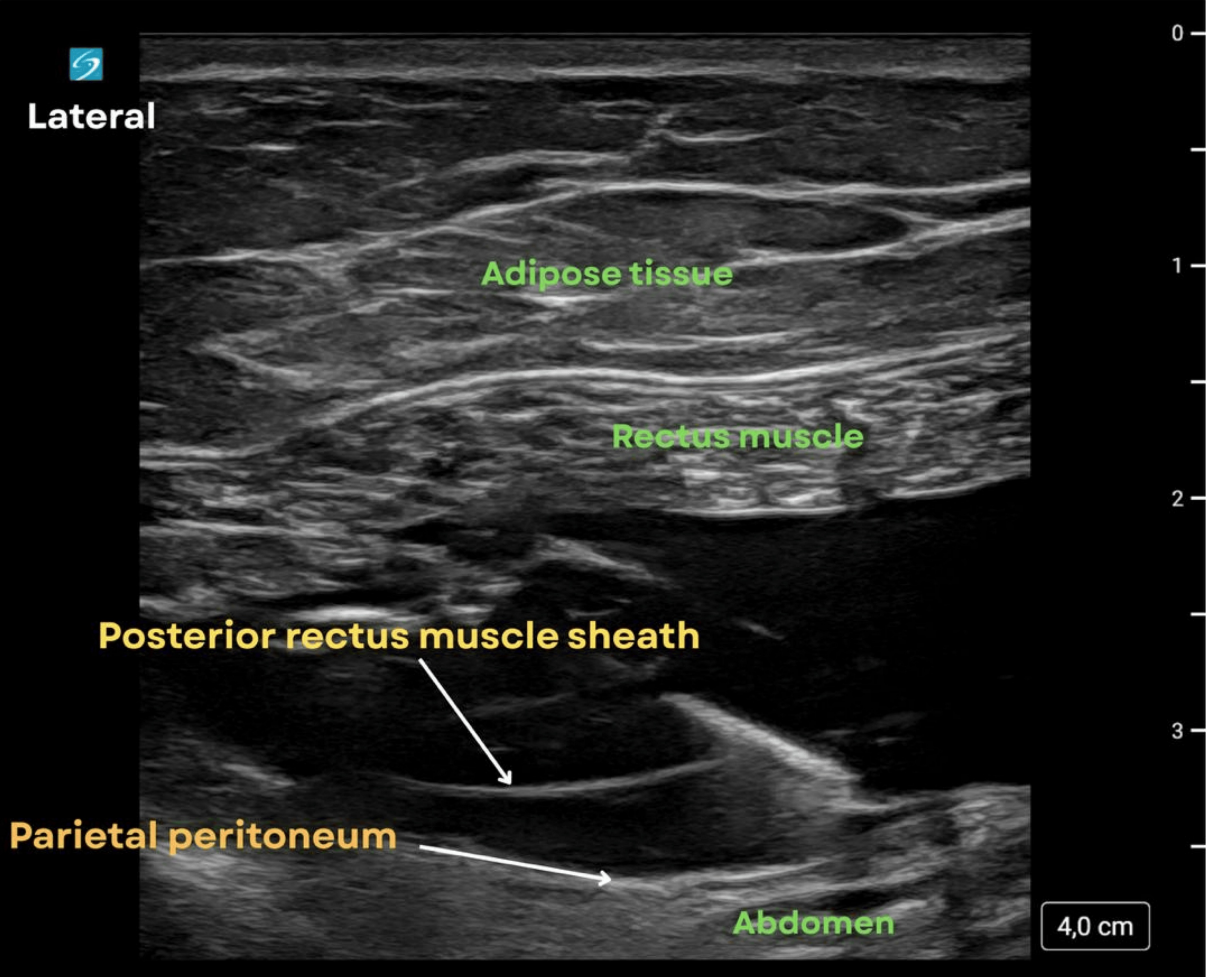
DRS block After an initial saline injection into the conventional rectus sheath plane to visualize the needle tip, the needle is advanced through the posterior rectus sheath into the preperitoneal plane. Injection of the local anesthetic opens the preperitoneal compartment, producing a characteristic V-shaped spread and displacing the parietal peritoneum deeper. Ultrasound orientation markers are shown within the image (lateral to the left, medial to the right; superficial at the top, deep at the bottom). DRS: deep rectus sheath

The bilateral DRS block was subsequently performed at the umbilical level, with the linear probe placed transversely over the rectus abdominis muscle. The needle was advanced from lateral to medial under direct ultrasound visualization until the deep plane of the rectus sheath was reached. First, normal saline was injected into the conventional rectus sheath plane to facilitate visualization of the needle tip. The needle was advanced through the posterior rectus sheath into the preperitoneal space, superficial to the parietal peritoneum/fascia transversalis complex. A total of 40 mL of ropivacaine 0.2% (120 mg) was administered bilaterally (20 mL per side), with the typical V-shaped spread of local anesthetic confirming correct needle-tip position within the interfascial plane and without complications (Figure 2).

Thirty minutes were allowed for block onset before proceeding with surgery, with dermatomal coverage extending from T6-7 to T12-L1 (pinprick and cold test). To improve comfort, sedation was induced with dexmedetomidine (loading dose 1 mcg/kg over 10 minutes, followed by a continuous infusion of 1.2 mcg/kg/h) in combination with ketamine 0.25 mg/kg/h, for a total administered dose of 28 mg over the course of the procedure, while maintaining a Richmond Agitation-Sedation Scale (RASS) score of −3 [10].

Laparoscopic cholecystectomy was performed without technical difficulties by the surgical team in 90 minutes, without complications, with the pneumoperitoneum pressure maintained at 10 mmHg. Hemodynamics remained stable throughout the procedure, with mean arterial pressure maintained around 65 mmHg and no episodes of bradycardia (defined as heart rate (HR) <50 beats/min). The patient breathed spontaneously throughout surgery, with oxygen supplementation (via nasal cannula at 4 L/min) and without the need for airway instrumentation. End-tidal CO₂ levels were < 42 mmHg. No intraoperative complications were recorded.

At the end of the procedure, dexmedetomidine infusion was stopped, and the patient was transferred to the intensive care unit for postoperative monitoring, breathing spontaneously with supplemental oxygen, and with residual sedation (RASS score of -2). The patient required 1,000 mL of crystalloids (administered during the fourth hour of ICU stay) and inotropic support with norepinephrine (0.1 μg/kg/min) from the sixth to the sixteenth hour of the ICU stay. Thereafter, norepinephrine was discontinued because hemodynamic stability had been achieved. Intravenous paracetamol was administered as part of the postoperative analgesic regimen (1 g three times daily). No rescue opioids were administered. Postoperative pain scores remained <3 on the Numeric Rating Scale. After 20 hours of postoperative monitoring, the patient (alert and calm) was transferred to the surgical ward, where her postoperative course remained uneventful (Table 2).

**Table 2:** Hemodynamics in ICU BP, HR, fluid resuscitation, and vasopressor support are reported from ICU admission to discharge. The patient developed transient hypotension at four hours, which responded to crystalloid infusion followed by temporary norepinephrine support, allowing hemodynamic stabilization and ICU discharge at 20 hours. ICU: intensive care unit, BP: blood pressure, HR: heart rate

Time	Hemodynamics	Intervention
0 h	BP 101/43 mmHg, HR 66 bpm	ICU admission
2 h	BP 109/50 mmHg, HR 70 bpm	-
4 h	BP 85/45 mmHg, HR 110 bpm	1,000 mL crystalloids
6 h	BP 115/60 mmHg, HR 115 bpm	Norepinephrine 0.1 μg/kg/min started
8 h	BP 110/50 mmHg, HR 99 bpm	Norepinephrine continued
12 h	BP 120/50 mmHg, HR 70 bpm	Norepinephrine continued
16 h	BP 110/60 mmHg, HR 66 bpm	Norepinephrine discontinued
20 h	BP 107/48 mmHg, HR 70 bpm	Discharged from ICU

She was discharged home on postoperative day 8, with a Clavien-Dindo classification of grade I (no complications) [11].

## Discussion

This case shows the successful use of a combined ITP and DRS block as the primary anesthetic technique for emergency laparoscopic cholecystectomy in a frail, high-risk elderly patient in whom both general and neuraxial anesthesia carried substantial risk.

The rationale for combining these two techniques lies in their complementary analgesic coverage. The ITP block, acting as a paravertebral-by-proxy technique, targets visceral afferents through potential migration of local anesthetic toward the thoracic paravertebral space via the superior costotransverse ligament. This mechanism, together with the anatomical basis and clinical applications of the different ITP block variants, has recently been summarized in a comprehensive narrative review of the technique [12]. The DRS block, in turn, provides analgesia through a distinct mechanism, acting directly on the parietal peritoneum, a richly innervated fascial plane contributing to abdominal proprioception and nociception. Nevertheless, targeting the peritoneum for postoperative analgesia is not a novel concept: intraperitoneal instillation of local anesthetic has previously been investigated as an alternative to systemic and even epidural analgesia after laparoscopic cholecystectomy, with studies demonstrating clinically meaningful pain relief and favorable pharmacokinetics with intraperitoneal ropivacaine [13], as well as postoperative pain control achieved through intermittent catheter-based instillation of ropivacaine into the peritoneal cavity [14]. The DRS block builds on this same anatomical rationale, targeting the parietal peritoneum from a preperitoneal fascial-plane ultrasound-guided approach rather than through direct intraperitoneal instillation, and this preperitoneal strategy has been further corroborated by a recent randomized controlled trial demonstrating reduced postoperative morphine consumption with laparoscopic-guided DRS block after laparoscopic cholecystectomy [8]. Taken together, the ITP block broadens dermatomal and visceral coverage through its paravertebral-directed spread. In contrast, the DRS block reinforces peritoneal and somatic analgesia through a distinct, fascial-plane mechanism. Although these effects may appear redundant, each block contributes a distinct dimension of pain control.

In our patient, this complementary coverage was clinically consequential rather than theoretical: her ASA IV status, septic presentation, hypoalbuminemia, and coagulopathy (INR 1.9) precluded neuraxial and paravertebral technique, leaving fascial plane blocks as the only locoregional option capable of providing surgical anesthesia, not merely postoperative analgesia, for a 90-minute laparoscopic procedure. The dermatomal spread achieved (T6-7 to T12-L1) proved sufficient to cover both the visceral and parietal components of the surgical stimulus, consistent with the combined mechanism described above. The stable intraoperative hemodynamics, the absence of complications, and the uneventful postoperative course (Clavien-Dindo grade I) observed in our patient are consistent with outcomes reported for other paravertebral-by-proxy strategies used as primary anesthesia in comparably high-risk surgical candidates [5,6], and support the feasibility of this combined approach even in the presence of active sepsis and a severe comorbidity burden that would ordinarily preclude both general and neuraxial anesthesia.

Equally central to the success of this approach was the sedation strategy. In the absence of a secured airway, adequate and closely monitored sedation is not merely supportive but essential to patient comfort, surgical conditions, and overall safety, requiring continuous oxygen therapy, vigilant clinical monitoring, and close coordination with the surgical team throughout the procedure. The combination of dexmedetomidine and ketamine was selected for their complementary sedative and analgesic properties, with complementary pharmacologic profiles. The dexmedetomidine regimen employed was not low, reflecting the depth of sedation required to maintain comfort throughout a 90-minute laparoscopic procedure; this was corroborated by the persistence of sedation on emergence, with the patient transferred to the intensive care unit still sedated (RASS -2) and requiring monitoring. However, she remained hemodynamically stable and continued to breathe spontaneously. Careful titration of both agents remains essential, particularly given the total volumes of local anesthetic required by the combined fascial-plane technique, to minimize the risk of local anesthetic systemic toxicity and other side effects, such as hypotension and bradycardia.

Beyond the analgesic rationale, this case underscores the critical importance of a coordinated, multidisciplinary team approach. Proceeding with a locoregional strategy as the primary anesthetic technique in a patient of this complexity required close cooperation with the surgical team, shared awareness of the contingency plan, and readiness to convert to general anesthesia at any point should the blocks prove insufficient or surgical conditions require it. This preparedness is not a peripheral detail but a structural component of the technique's safety.

Finally, this combined fascial plane block approach should be regarded as exploratory rather than a routine alternative to general or neuraxial anesthesia [15]. It is technically demanding, requires advanced ultrasound-guided regional anesthesia skills, and is associated with a non-negligible learning curve [16]. Its use should currently be reserved for carefully selected, high-risk patients in whom conventional techniques are contraindicated, ideally within centers experienced in fascial plane blocks. Further prospective studies are needed to confirm reproducibility, define optimal dosing, and establish the role of this combined technique within the broader landscape of locoregional anesthesia and fascial plane blocks for high-risk abdominal surgery.

## Conclusions

In our high-risk elderly patient with gangrenous cholecystitis, septic status, and a deranged coagulation profile precluding neuraxial anesthesia, a combined ITP and deep rectus sheath block, supported by dexmedetomidine-ketamine sedation, allowed emergency laparoscopic cholecystectomy to be completed safely, with stable intraoperative hemodynamics and an uneventful postoperative course. This case supports the feasibility of combining paravertebral-by-proxy and preperitoneal fascial plane blocks as a primary anesthetic strategy in frail patients unsuitable for conventional approaches, while underscoring the need for further clinical experience to define its reproducibility and optimal role in this population.
